# The Effects of Teacher Rewards and Their Types on Preschool Children’s Selective Trust

**DOI:** 10.3390/bs15060804

**Published:** 2025-06-12

**Authors:** Weihai Tang, Yuqian Du, Rubo Zhong, Chunhui Qin, Xiping Liu

**Affiliations:** 1 School of Sociology, University of Sanya, Sanya 572022, China; twhpsy@126.com; 2 Key Research Base of Humanities and Social Sciences of the Ministry of Education, Academy of Psychology and Behavior, Tianjin Normal University, Tianjin 300387, China; duyuqian710@126.com (Y.D.); 13624915762@163.com (C.Q.); 3 School of Preschool and Special Education, Kunming University, Kunming 650214, China; zhongrubo@163.com

**Keywords:** reward, selective trust, material reward, praise

## Abstract

Children acquire much of their knowledge through trusting others’ testimony, particularly that of teachers. They not only tend to trust their teachers but also imitate behaviors that teachers reward. However, it remains unclear if they show selective trust in those who provide such rewards. This study, therefore, examined how teachers’ rewards to other children and the types of these rewards influence the selective trust of preschoolers. In Study 1, 162 preschoolers from junior, middle, and senior classes watched videos of a teacher giving verbal and material rewards, while another provided neutral feedback. Then, children chose which teacher to trust in a novel object-naming task. The results showed that all preschoolers preferred to trust teachers who offered rewards compared to those who did not. Moreover, junior-class children displayed the highest level of selective trust among the preschoolers. In Study 2, 176 preschoolers judged which teacher to trust, one offering material rewards and the other verbal praise. The results showed senior-class girls preferred teachers with material rewards more than senior-class boys and middle-class girls. These findings indicate that preschoolers can assess teachers’ trustworthiness based on rewards and are more sensitive to material rewards than to verbal praise when accepting information from teachers.

## 1. Introduction

Piagetian theory posits that children are autonomous agents actively constructing their understanding of the world (Piaget, 1954), whereas much of human knowledge lies beyond the reach of their direct experience or observation (Harris et al., 2006). For example, children often encounter intangible concepts, such as historical figures they can never meet, unseen landscapes, and various abstract principles (Harris et al., 2012). In these cases, they must rely on information conveyed by others (Corriveau et al., 2009). To ensure the authenticity of such information, children need to discern the reliability of informants, which requires them to trust others selectively. Initially, selective trust refers to the children’s ability to choose which informants to trust based on their past accuracy (Koenig et al., 2004). It is also defined as the process by which children use various cues to assess informants’ attributes and subsequently make selective trust decisions (Harris et al., 2012; Mills, 2013). In the process of selective trust, children may form a preference for certain informants, leading to a global impression that influences whether they accept the information provided (Bascandziev & Harris, 2014; Fusaro et al., 2011). Alternatively, they may engage in rational evaluation of the informants’ capabilities and knowledge, judging the reliability of their information accordingly (Hermes et al., 2015; Lutz & Keil, 2002). Based on Hermes et al.’s (2018) dual-process theory, the first approach reflects heuristic selective trust, while the second represents a selective trust based on trait reasoning.

Children demonstrate selective trust in information sources, which is significantly influenced by the identity of the source, particularly when the source is a teacher. For example, three- to five-year-old children are more likely to change their views when a teacher expresses disagreement with their opinion compared to when a peer disagrees with them (Ma et al., 2017). Furthermore, when teachers and the internet offer conflicting information, elementary school children tend to place greater trust in the teacher’s input rather than that of the internet (Guerrero et al., 2020; F. Wang et al., 2019). When children are presented with scientific explanations from different sources (teacher vs. book) and of varying qualities (circular vs. non-circular explanations), five-year-old children tend to trust the teacher as a source of knowledge, whereas children aged seven and above rely more on the quality of the explanation. This suggests that younger children primarily rely on the teacher’s identity to form trust judgments (Wu et al., 2024). These results indicate that children tend to trust teachers’ information more than that of other sources. Although previous studies have primarily focused on the effects of the overall identity of teachers and other informants on selective trust, an open question remains: do children place greater trust in teachers who provide rewards to other children compared to those who do not? This study, therefore, aims to shed light on this issue.

Selective trust plays a crucial role in children’s behavioral decision-making, as it not only determines from whom children are willing to seek information but also significantly influences their behavioral choices. For example, studies have found that children aged 2–4 continue to select and imitate an informant’s actions even when the informant uses a functionally inferior tool, provided that the informant emphasizes its design advantages (DiYanni & Kelemen, 2008). Furthermore, children showed greater trust in and imitation of individuals whose actions demonstrated clear causal efficacy compared to those whose behaviors lacked explicit causal explanations (Wilks et al., 2016). These findings underscore the critical role of selective trust in shaping children’s behavioral patterns, as they prioritize informants whose actions align with perceived reliability and causal logic. To further explore the relationship between selective trust and children’s behavior, it is necessary to consider the connection between selective trust and rewards, as well as the various types of rewards offered by teachers.

### 1.1. How Teachers’ Rewards Shape Children’s Behavior

Children are more likely to exhibit the behaviors that their teachers have rewarded. For example, the principle of vicarious reinforcement suggests that when children observe others being rewarded for certain behaviors, they may infer that they will receive similar rewards if they perform those same behaviors, thereby increasing the likelihood of engaging in them. This is further supported by the principle of reciprocity in social exchange theory (Bandura, 1965; Homans, 1958). Additionally, research has found that praise can be a strong motivator for children to repeat specific behaviors, and meaningful rewards can enhance their persistence in tasks and further encourage them to engage in the rewarded behaviors (Alvarez & Booth, 2014; Brophy, 1981). Van Mastrigt et al. (2024) show that children will adjust their drawing behavior based on reward feedback and display strong persistence. This suggests that meaningful rewards (such as positive feedback) can effectively enhance children’s motivation and prompt them to engage in rewarded behaviors actively. Grubliauskiene et al. (2012) found that when children received tangible rewards and praise for choosing healthy food, they were more likely to continue making healthy choices in the future. These findings collectively support the view that children are more likely to engage in behaviors that are recognized and rewarded by their teachers.

Children also tend to imitate individuals who have been praised by their teachers. For example, Schoneveld and Brummelman (2023) found that children perceive students who are praised as more hardworking and are more likely to imitate such students. In addition, children are sensitive to social information, and teacher praise can be seen as an important social cue that may influence children’s choices of whom to imitate (Burdett et al., 2018; Z. Wang & Meltzoff, 2020). Legare and Nielsen (2015) proposed that imitation is an important component of cultural learning, and teacher praise may be a key factor in this process, guiding children to imitate certain behaviors. These studies suggest that teachers’ rewards influence children’s behaviors.

As we can see, teachers’ rewards play a significant role in influencing children’s behavior patterns through the mechanism of learning and imitation. However, children’s cognitive process regarding teachers’ rewards is not limited to just imitating the rewarded behaviors. They also start to reason about the identity of the teacher who provides the rewards. This leads to an interesting exploration of how children reason about teachers’ behavior in providing rewards to other children, particularly in the context of selective trust.

### 1.2. The Relation Between Teachers’ Reward Behaviors and Selective Trust

How does children’s selective trust operate when they encounter teachers who share the same authoritative identity but differ in their behaviors about rewards? There are several possibilities regarding the inferences that children make about teachers’ rewards to other people.

The first explanation is the social exchange theory (Homans, 1958). Rooted in the principle of reciprocity (Homans, 1958), this theory posits that children evaluate teachers’ fairness by observing whether rewards align with behavioral merit. For example, research has found that students form judgments about teachers by assessing whether their attitudes and behaviors are fair (Gingo, 2017; Kaufman & Killen, 2022; Tomul et al., 2012). Children who feel unfairly treated due to their background or personal beliefs are more likely to perceive the teachers’ reward and punishment system as biased (Kaufman & Killen, 2022). Conversely, children who observe teachers appropriately rewarding positive behaviors and withholding rewards or imposing consequences for misconduct are more inclined to regard the teacher as fair and impartial (Tomul et al., 2012). Through their inferences about the teacher’s reward practices, children form judgments regarding the teacher’s trustworthiness. These trust judgments, in turn, may influence children’s decisions about whether to trust the teacher.

The second explanation is social signal processing (Pentland, 2007). Teachers’ reward-giving behaviors act as social signals that children interpret to infer hidden attributes. Rewards for prosocial acts (e.g., sharing) may signal teachers’ goodwill (Ambady & Rosenthal, 1993), while rewards for correct behaviors signal competence in discerning appropriate actions (Kim et al., 2023). On the other hand, if teachers fail to reward those who display correct or appropriate behaviors, children may infer that the teacher lacks the relevant ability (Kim et al., 2023).

The third possible explanation is social learning theory. According to social learning theory, individuals learn by observing the behaviors of others (models) and the outcomes of those behaviors (Bandura, 1965, 1977, 1985). When teachers reward peers, children engage in vicarious reinforcement, associating rewarded behaviors with positive outcomes and inferring that imitating these behaviors may yield similar rewards. For example, if a child sees a peer receive praise (a reward) from the teacher for answering a question correctly, they will associate this behavior with the reward (Apps et al., 2015). This observational learning not only influences the child’s behavioral tendencies but also affects their perceptions of the teacher. As the provider of rewards, the teacher’s actions are interpreted by children as a social signal (Gingo, 2017; Kaufman & Killen, 2022; Tomul et al., 2012). If the teacher’s rewards are perceived as fair, reasonable, and closely linked to positive behaviors, children are likely to view the teacher as highly credible. Recent studies found that, in social reinforcement learning, others’ behaviors act as “pseudo-rewards” to drive imitation via value shaping. Demonstrators’ actions affect learners’ value functions, like rewards. Observing others being rewarded for certain behaviors leads individuals to infer their value and expect similar rewards for themselves if they imitate (Najar et al., 2020).

These theories all posit that the act of teachers providing rewards may influence children’s selective trust in teachers. Teachers who provide rewards to others are perceived as more trustworthy.

### 1.3. Types of Teachers’ Rewards

Given that teachers’ reward behavior may influence children’s selective trust, it is necessary to investigate further whether the types of rewards teachers provide to other children affect children’s selective trust. Rewards are defined as positive evaluations of students’ academic performance and good behavior. They can enhance the cognitive control and task accuracy of children. For example, social rewards, such as praise, serve not only as motivational tools but also directly improve performance, demonstrating their dual role in both recognition and behavioral reinforcement (Jin et al., 2024).

Rewards can be divided into material rewards (e.g., physical gifts) and spiritual rewards (e.g., verbal praise) (Sun, 2008). Material rewards refer to tangible items or objects that are given to individuals as an incentive or reinforcement for their behavior. They include items such as toys, snacks, money, or other physical goods (Eisenberger & Cameron, 1996). Spiritual rewards, also known as intangible rewards, are non-material incentives designed to fulfill an individual’s psychological needs. They often involve recognition, praise, honor, or a sense of accomplishment (Brophy, 1981; Jin et al., 2024).

Numerous studies have shown that material rewards and spiritual rewards have distinct impacts on children’s behavior. For example, by the age of four, children can distinguish between praise and material rewards (Danner & Lonky, 1981; Zhao, 2019). Moreover, recognition and affirmation from teachers are highly attractive to young children, and both forms of praise can provide positive emotional experiences (Berridge & Robinson, 1998; Cameron & Pierce, 1994; Godfrey et al., 2023; Shen, 2023). Effective praise can encourage and support children based on their effort, helping to establish a friendly relationship between teachers and students (Brophy, 1981; Weisz, 1977). Material rewards, such as stickers, toys, monetary feedback, and treats, are also highly appealing to young children. However, for older children, material rewards tend to be more attractive than spiritual rewards (Ding et al., 2017; Kohls et al., 2009; Sun, 2008). For example, research has found that children in the senior kindergarten class are more sensitive to material rewards (Sun, 2008), whereas older children, around the age of 8, show a stronger response to monetary feedback than to social feedback (Ding et al., 2017; Kohls et al., 2009). Additionally, the positive reinforcement effect of rewards has been documented (Apps et al., 2015; Cameron & Pierce, 1994), with middle-class children showing increased persistence under material reward conditions (Y. J. Wang, 2018).

Additionally, there are gender differences in children’s sensitivity to rewards. For example, studies on gender differences in the processing of rewards and punishments in contexts of monetary and social feedback (such as smiles or frowns) have shown that girls show higher sensitivity and flexibility in feedback processing, while boys exhibit a lower attention to and processing speed of feedback (Crowley et al., 2013; Ding et al., 2017). However, the impact of rewards on children’s behavior is not always positive. Some studies have shown that material rewards can reduce children’s sharing behavior (Ulber et al., 2016) and lead to an “over-justification effect,” where children’s behavioral tendencies decrease after the withdrawal of rewards (Smith & Pittman, 1978). Moreover, Fantuzzo et al. (1991) found that elementary school children generally prefer verbal praise over material rewards.

These findings suggest that material rewards and verbal praise have different impacts on children at different ages. Material rewards may be perceived as more “instrumental” since they provide direct, tangible value, while verbal praise may be seen as more social, as it emphasizes the relationship between teachers and children (Brophy, 1981). Thus, children may make different judgments about teachers’ trustworthiness based on the type of teachers’ reward.

### 1.4. The Current Research

Given this, the current study aims to fill the gap in the literature by examining how teachers’ reward behaviors and the types of rewards influence preschoolers’ selective trust. Specifically, this study investigates whether teachers’ reward behaviors toward other children trigger selective trust in these teachers and whether different types of rewards (material vs. verbal) elicit different degrees of selective trust.

In this study, we designed two experiments using a conflicting information source paradigm (Koenig et al., 2004) to explore how teachers’ reward behaviors influence preschool children’s selective trust. The paradigm included two phases: familiarization and test. During the familiarization phase, children watched animated videos featuring two teachers. In Experiment 1, one teacher provided both verbal praise and material rewards for children’s actions, while the other teacher offered neutral feedback. In Experiment 2, one teacher provided material rewards, and the other offered only verbal praise. These contrasting behaviors allowed children to form initial impressions of the teachers’ reliability based on their reward practices. In the test phase, children encountered novel objects and were asked to decide which teacher to ask for information (inquiry task) or whose label to endorse (endorsement task). These tasks captured distinct aspects of selective trust: the inquiry task reflected children’s preference for reliable sources when seeking new information, while the endorsement task revealed their ability to evaluate and choose between conflicting claims based on past reliability. By using this paradigm, we aimed to assess whether children’s selective trust is influenced by the type of reward and whether this effect varies with age and gender.

Hermes et al. (2018) proposed a dual-process model that provides a theoretical framework for understanding children’s selective trust, positing that it emerges from the interaction of two cognitive systems: a heuristic (intuitive) system and an analytic (logical) system. These processes evolved during early development, coexisting with children’s reliance on either system, which is influenced by cognitive resources, knowledge, and task demands. Initially, children defaulted to heuristic strategies, generating quick trust or distrust responses through superficial cues. As cognition matured, analytic processing gradually complemented heuristics, enabling nuanced trust decisions. Ronfard et al. (2019) observed children’s dynamically adjusted trust by tracking informants’ accuracy, reflecting shifting cognitive strategies. Research has shown that children as young as three prioritize irrelevant cues (e.g., appearance) when selecting informants (Bascandziev & Harris, 2014). By employing this theory, we aim to explore whether teachers’ rewards, especially material rewards, can serve as potent heuristic cues for young children, influencing their selective trust even in the absence of explicit reliability information.

In summary, this study hypothesizes that children tend to trust teachers who provide rewards when learning uncertain new information, and this selective trust is related to gender and age. Different types of rewards have different impacts on children’s selective trust. Specifically, children tend to trust teachers who provide material rewards when learning uncertain new information, and this selective trust is related to gender and age, and a dual-process model may influence it.

## 2. Study 1: The Impact of Teachers’ Reward Behavior on Preschool Children’s Selective Trust

### 2.1. Method

#### 2.1.1. Participants

The sample size was calculated by using G*Power 3.1.9.2 with a medium effect size of 0.25, an alpha value of 0.05, and a statistical power of 0.8. The results showed that a total sample size of 162 or more was determined. The participants were 169 preschoolers from three kindergartens, aged between 3 and 7 years (*M* = 4 years, 9 months; *SD* = 10.85 months; range: 3 years, 2 months to 6 years, 4 months; 76 girls, 93 boys). They were divided randomly into three groups, including junior class, middle class, and senior class (see Table 1).

#### 2.1.2. Design

A mixed experimental design of 2 (task type: inquiry, agreement) × 2 (gender: male, female) × 3 (grade: junior class, middle class, senior class) was used, with the task type as the within-subject variable and gender and grade as between-subject variables. The dependent variable was the subjects’ trust in the reward-giving teacher. Selective trust indicators were divided into two metrics: inquiry rate and agreement rate. The inquiry rate and agreement rate were mainly used to examine whether selective trust occurred. The inquiry rate was the ratio of the number of inquiries made to the reward-giving teacher to the total number of inquiries, and the agreement rate was the ratio of the number of agreements to the novel object names proposed by the reward-giving teacher to the total number of agreement trials. Selective trust degree indicators were divided into two metrics: inquiry degree and agreement degree. Both were the inquiry rate and agreement rate minus 0.5, with 0.5 being the chance level. The selective trust degree indicators mainly refer to the extent of deviation from the chance level.

Control of irrelevant variables: The teacher informants were identical except for the color of the paper-cut silhouette; the teacher praise and reward objects were the four most frequently used by teachers; the children’s activities were the four selected by teachers as the most reflective of children’s sense of achievement; the position of the teacher informant photos was random; and the presentation order of the novel object images was random.

#### 2.1.3. Materials

The teacher reward videos were produced using WanCai Animation Master 2.7.7, and the novel object experiment procedure was compiled using E-Prime and presented on a 14-inch Windows laptop computer. Data collection was completed using an individual testing method. The experimental materials included teacher informants, teacher reward stories, novel object names, and artificial pseudo-words.

**Figure 1:**
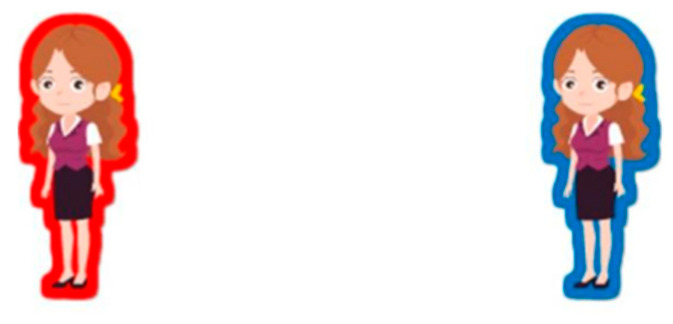
Teacher images (left, “Teacher Red,” and right, “Teacher Blue”). This figure was created using materials from Animiz Information Technology Co., Ltd, Guangzhou, China. (www.animiz.cn).

1. Informants. This study used two paper-cut cartoon images of teacher figures as informants, with the paper-cut silhouettes of the teachers being red and blue, respectively, and referred to as “Teacher Red” and “Teacher Blue.” The teacher’s images can be seen in Figure 1.
2. Teacher praise and reward objects and children’s activity evaluation: First, nine teachers were asked to complete a teacher praise and reward survey, using an open-ended questionnaire to select the most frequently used praise and reward objects in kindergarten. Based on this, the top 10 most frequently mentioned targets of praise and rewards were selected to form the Preschool Teacher Praise and Reward Survey (see Appendix A). Forty-one teachers were asked to complete the Preschool Teacher Praise and Reward Survey. Finally, the four most frequently used verbal praises, four reward objects, and the four activities that best reflect children’s sense of achievement after completion were selected. The survey results are presented in Appendix B. The experimental video, adapted from the stories in Kamins and Dweck (1999), consists of four activities, allowing children to become familiar with the teachers’ behavior and make judgment choices. In the experimental video, the reward-giving teacher is shown praising the child and then giving a physical reward object after the child completes the activity. The non-reward-giving teacher provides neutral feedback by simply stating the facts without offering any rewards, ensuring linguistic balance and phonetic rationality while avoiding any emotional connotations. For example, in the block-building activity, neutral feedback is “Lele, you have finished building.” In the craft-making activity, neutral feedback is “Lele, you have completed it.”
3. Equivalence evaluation of novel objects and corresponding artificial pseudo-word names: Previous research has introduced a database of novel objects and names (NOUN database) which can be applied to developmental psychology and other related fields, such as perception and visual memory (Horst & Hout, 2016). This study selected novel objects from this database. Thirty children who did not participate in the formal experiment, from the junior and middle classes, were asked to evaluate the objects. The children watched the novel objects and answered whether they recognized them or not. An analysis was conducted to calculate the number of children who did not recognize the novel objects and the ratio of children who chose the artificial pseudo-word names for the novel objects. A goodness-of-fit test was performed, and four sets of novel objects with non-significant test results were selected as official experimental materials.

#### 2.1.4. Procedure

The experiment was completed in a quiet room, lasting approximately 7–12 min. The specific experimental process is as follows.

1. Collection of participant information

A harmonious relationship is established with the children and relevant information collected, such as their names, grades, and genders.

2. Familiarization process

**Figure 2:**
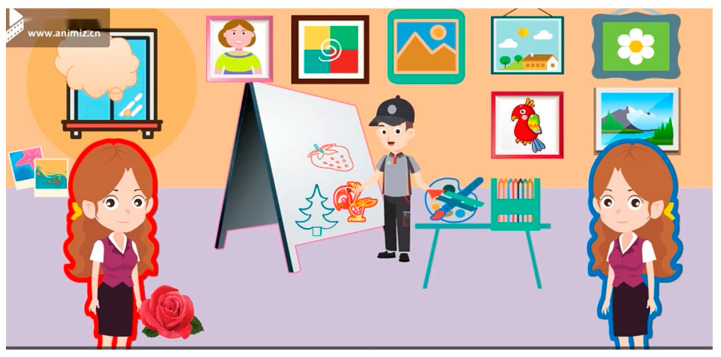
Video screenshot: teacher giving Lele a reward (reward mentioned in the video). This figure was created using materials from Animiz Information Technology Co., Ltd, Guangzhou, China. (www.animiz.cn).

In the familiarization phase, Video 1 is watched to familiarize the children with the different behaviors of the two teachers in the four activities. Teacher Red praised and rewarded “Lele” in all four activities, while Teacher Blue only provided neutral feedback in the four activities. The process mainly consists of two steps. The video frame is shown in Figure 2, and Lele is the little boy in the video frame.

Step 1: Introduce the informants. The experimenter tells the children, “Hello, kids! Today we are going to watch an animated cartoon. There is a little friend named Lele, and two teachers appear in the cartoon: Teacher Red and Teacher Blue. Let us see what happens in the cartoon!”

Step 2: Ask about the teacher’s characteristics. The children are asked, “Do you remember what Teacher Red said? What did Teacher Blue say? Which teacher praised and rewarded Lele? Which teacher did not reward Lele?” The children must answer correctly before proceeding to the formal experiment.

3. Formal experimental procedure

The formal experiment is divided into four trials, each with different novel objects and names, with the same specific experimental operations.

Step 1: Introduce the experiment. The experimenter shows the children the novel object image presented in the center of the notebook and asks if they recognize the object. First, they ask the children, “Hello, kids! Let us play a little game. There will be some things we do not know about coming up. I do not know what they are. Can you help me?” The novel object is presented on the screen and they are asked again, “Have you seen this thing before? Do you know what it is?” Then, they record the children’s reactions. If the child gives the name of the object, they tactfully tell them that the name they provided is incorrect; if the child answers that they do not know, they proceed to the next step.

Step 2: Inquiry task. The images of the two teacher informants are presented on the screen and the experimenter asks the child, “Both teachers know what this thing is called. Which teacher would you like to ask to tell you the name of this object?” They observe and record the child’s verbal or pointed choice, and the experimenter presses the key to record the child’s choice.

Step 3: The informants provide different names for objects. First, the child is told, “Both teachers told us what this thing is called, but only one teacher gave the correct name. Let us listen to what both teachers said.” The object name is presented directly below the two teacher informants, and the experimenter points to the informants one by one and states the object name.

Step 4: Agreement task. Both the two teacher informants and the novel object are presented on the screen simultaneously. The child is asked, “Which teacher do you think is right about the name?” The child’s verbal or pointed choice is recorded, and the experimenter presses the key to record the child’s choice.

4. End of the experiment

The child’s behavior is affirmed and praised, and the experimenter gives the child a sticker as a reward.

### 2.2. Results

Data analysis and processing were conducted using Microsoft Excel 2016 and SPSS 22.0 software.

#### 2.2.1. Selective Trust of Preschool Children in Reward-Giving Teachers

A one-sample *t*-test was performed on the inquiry rate and agreement rate of preschool children towards reward-giving teachers as dependent variables, comparing them with the random level of 0.5, to examine whether preschool children exhibit selective trust towards reward-giving teachers. As shown in Table 2, the inquiry and agreement rates of preschool children towards reward-giving teachers were both significantly higher than the random level, indicating that preschool children demonstrated selective trust towards reward-giving teachers.

#### 2.2.2. Selective Trust Characteristics of Gender and Grade in Children

A repeated measures ANOVA was conducted on the 2 (task type: inquiry, agreement) × 2 (gender: male, female) × 3 (grade: junior class, middle class, senior class) experiment with the inquiry degree and agreement degree of children towards reward-giving teachers as dependent variables, to investigate the characteristics of selective trust towards reward-giving teachers among preschool children of different genders and grades.

The results showed that the interaction effects of task type, gender, and grade were not significant, *F* (2, 163) = 0.229, *p* = 0.795; the interaction effect of task type and gender was not significant, *F* (1, 163) = 1.45, *p* = 0.23; the interaction effect of task type and grade was not significant, *F* (2, 163) = 0.439, *p* = 0.645; and the interaction effect of gender and grade was not significant, *F* (2, 163) = 1.322, *p* = 0.27. The results indicate that the development of selective trust towards reward-giving teachers among preschool children of different genders and grades is consistent. The main effect of task type was not significant, *F* (1, 163) = 0.121, *p* = 0.729, suggesting that there was no significant difference in the degree of selective trust towards reward-giving teachers between inquiry and agreement tasks among preschool children, indicating that teacher rewards have a certain effect on preschool children’s selective trust. The main effect of gender was not significant, *F* (1, 163) = 1.012, *p* = 0.316, indicating that there was no difference in the degree of selective trust towards reward-giving teachers among children of different genders. The main effect of grade was significant, *F* (2, 163) = 3.817, *p* = 0.024 < 0.05, $\eta_{p}^{2}$ = 0.045. Further multiple comparisons (LSD) revealed that the selective trust degree of children in the junior class towards reward-giving teachers was significantly higher than that of children in the senior class (*p* = 0.02 < 0.05), while there were no significant differences between junior- and middle-class children (*p* = 0.051) and between middle- and senior-class children (*p* = 0.728). This suggests that children in the junior class are more sensitive to the rewards of teachers than those in the senior class. Based on this, we also supplemented the analysis with a more stringent method. The results of the multiple comparisons (Bonferroni) showed that there were no significant differences in the trust levels of children from the senior, middle, and junior classes towards teachers who provided rewards (senior vs. middle class: *p* > 0.999, senior vs. junior class: *p* = 0.06, middle vs. junior class: *p* = 0.154).

### 2.3. Discussion

Experiment 1 primarily investigated the effect of different teacher types on children’s selective trust. The teacher types were divided into reward-giving teachers and non-reward-giving teachers. Reward-giving teachers provided both material rewards and praise, while non-reward-giving teachers only provided neutral feedback. The results showed that preschool children exhibited selective trust towards reward-giving teachers, consistent with previous research findings that rewards have a positive motivational effect on children’s behavior (Fantuzzo et al., 1991).

The study also found that the development of selective trust towards reward-giving teachers among preschool children of different genders and grades was consistent. However, the degree of selective trust towards reward-giving teachers varied among preschool children. Multiple comparisons were conducted using both the Least Significant Difference (LSD) and Bonferroni correction methods. While the Bonferroni method (which strictly controls family-wise error rate) yielded more conservative results, we opted to report the LSD findings for discussion, as this approach provides higher statistical power and is more sensitive to detecting potential effects, particularly given the exploratory nature of certain analyses. Children in the junior class showed the strongest trust in reward-giving teachers, while children in the middle and senior classes showed the same level of trust, indicating that junior-class children are the most sensitive to the rewards of teachers. Since junior-class children have just entered kindergarten and are less familiar with the kindergarten teachers compared to middle- and senior-class children, they have fewer interactions with the teachers. Currently, the teachers’ proactive rewards for the children’s completion of activities make it easier for the children to develop a greater sense of affection and trust towards the teachers who provide rewards. Previous research has found that preschool children develop a sense of dependence on adults who provide rewards (Weisz, 1977). The provision of rewards increases the interaction between teachers and children. As junior-class children are younger and their status is not yet stable, they are more likely to develop a sense of trust towards teachers who provide rewards. Middle- and senior-class children, who have spent more time with their teachers, have developed a certain familiarity with them. They also show selective trust towards reward-giving teachers, but the degree of trust is not as high as that of junior-class children. Additionally, the dual-process theory proposed by Hermes et al. (2018) may also explain this result. Judging the reliability of information based on whether the teacher provides rewards is a heuristic judgment, not a rational inference. The higher the children’s trust, the lower the rational component, and as children’s cognitive abilities develop, the rational component increases, and thus the degree of trust decreases.

The rewarding behavior of teachers influences the selective trust of preschool children, so what about the specific material rewards and praise? How do they affect children’s selective trust, and what are their characteristics and developmental patterns? These questions are the focus of Study 2.

## 3. Study 2: The Impact of Teachers’ Reward Types on Preschool Children’s Selective Trust

### 3.1. Method

#### 3.1.1. Participants

Using G*Power3.1.9.2 to calculate the sample size, with a medium effect size of 0.25, an alpha value of 0.05, and a statistical power of 0.8, a total sample size of 162 or more was determined. To ensure the reliability of the experimental results, 176 participants were selected from three kindergartens who did not participate in Experiment 1, aged between 3 and 7 years (*M* = 4 years, 8 months, *SD* = 11.13 months, range: 3 years, 1 months to 6 years, 7 months, 85 girls, 91 boys). They were divided randomly into three groups, including junior class, middle class, and senior class (Table 3).

#### 3.1.2. Design

A mixed experimental design of 2 (task type: inquiry, agreement) × 2 (gender: male, female) × 3 (grade: junior class, middle class, senior class) was used, with the task type as the within-subject variable and gender and grade as between-subject variables. The dependent variable was the subjects’ trust in the material reward teacher. The indicators of selective trust and the control of irrelevant variables were the same as in Experiment 1.

#### 3.1.3. Materials

The types of teacher rewards were different; the material reward teacher only provided physical rewards and expressed them through actions, while the praise teacher only verbally praised the children without physical rewards. The rest was the same as in Experiment 1.

#### 3.1.4. Procedure

The procedure was essentially the same as in Experiment 1, with only the familiarization process differing.

During the familiarization phase, children watched Video 1 to become familiar with the different behaviors exhibited by the two teachers in the four activities. Teacher Red praised and rewarded Lele in all four activities, while Teacher Blue only provided neutral feedback in the four activities. The process mainly consists of two steps.

Step 1: Introduce the informants. The experimenter tells the children, “Hello, kids! Today we are going to watch an animated cartoon. There is a little friend named Lele, and two teachers appear in the cartoon: Teacher Red and Teacher Blue. Let us see what happens in the cartoon!”

Step 2: Ask about the teacher’s characteristics. The children are asked, “Do you remember what Teacher Red said? What did Teacher Blue say? Which teacher gave Lele a prize? Which teacher did not give Lele a prize?” The children must answer correctly before proceeding to the formal experiment.

### 3.2. Results

The data processing method was the same as in Experiment 1.

#### 3.2.1. Selective Trust of Preschool Children in Material Reward-Giving Teachers

A one-sample t-test was performed on the inquiry rate and agreement rate of preschool children towards material reward-giving teachers as dependent variables, comparing them with the random level of 0.5. It was found that the inquiry rate and agreement rate of children in the junior class towards material reward-giving teachers were not significantly different from the random level, indicating that junior-class children did not exhibit selective trust towards material reward-giving teachers. In the inquiry task, children in the senior class all showed selective trust towards the material reward-giving teachers. In the agreement task, boys and girls in the middle class and girls in the senior class also showed selective trust towards material reward-giving teachers. Overall, not all preschool children exhibited selective trust towards material reward-giving teachers.

#### 3.2.2. Selective Trust Characteristics of Gender and Grade in Middle- and Senior-Class Children

Since some children in the middle and senior classes showed selective trust towards material reward-giving teachers, only the characteristics of selective trust towards material reward-giving teachers among children in the middle and senior classes were examined. A 2 (grade: middle class, senior class) × 2 (gender: male, female) × 2 (task type: inquiry, agreement) repeated measures ANOVA was conducted, with gender and grade as independent variables, and the inquiry degree and agreement degree of children towards material reward teachers as dependent variables.

**Figure 3:**
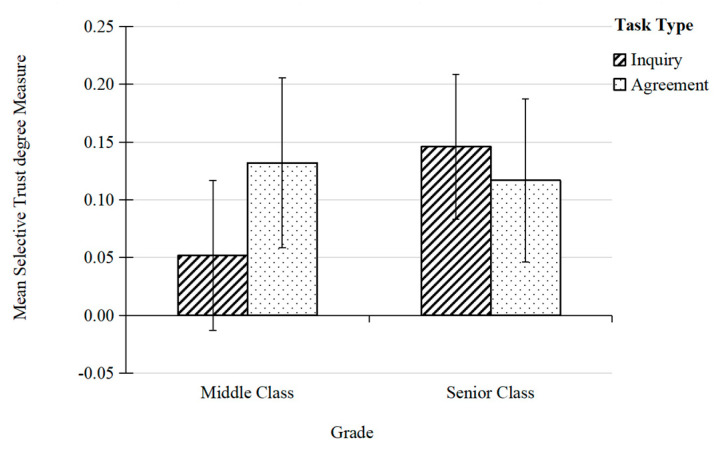
Interaction between task type and grade. Error bars represent the 95% confidence interval (95% CI).

The results of the ANOVA indicated that the interaction effects of task type, grade, and gender were not significant, *F* (1, 109) = 0.433, *p* = 0.512. The interaction effect of task type and gender was not significant, *F* (1, 163) = 0.399, *p* = 0.529. The interaction effect of task type and grade was significant, *F* (1, 109) = 3.994, *p* = 0.048 < 0.05, $\eta_{p}^{2}$ = 0.04 (Figure 3).

Further simple effect analysis of the data in Figure 3 revealed the following:

In the inquiry task, there was a grade difference in children’s selective trust towards teachers with material rewards, *F* (1, 109) = 4.272, *p* = 0.041 < 0.05, $\eta_{p}^{2}$ = 0.04. Children in the senior class showed significantly higher inquiry rates towards teachers with material rewards than those in the middle class. In the agreement task, there was no grade difference in children’s selective trust towards teachers with material rewards, *F* (1, 109) = 0.089, *p* = 0.765. This suggests that when inquiring about the name of the object, children in the senior class were more sensitive to material rewards.

Children in the middle class showed significant differences in their selective trust towards teachers with material rewards across different task types, *F* (1, 109) = 4.103, *p* = 0.045 < 0.05, $\eta_{p}^{2}$ = 0.04. Children in the middle class exhibited higher selective trust towards teachers with material rewards in the agreement task compared to the inquiry task, while children in the senior class did not show a difference in selective trust based on task type, *F* (1, 109) = 0.605, *p* = 0.438. This indicates that children in the senior class consistently trusted teachers with material rewards, regardless of whether they were inquiring about the object’s name or agreeing with the name provided by the teacher.

The interaction between grade and gender was significant, *F* (1, 109) = 7.241, *p* = 0.008 < 0.01, $\eta_{p}^{2}$ = 0.06. Further simple effect analysis revealed the following:

There was no gender difference in the selective trust of children in the middle class towards teachers with material rewards, *F* (1, 109) = 3.244, *p* = 0.074, while there was a gender difference in the selective trust of children in the senior class towards teachers with material rewards, *F* (1, 109) = 4.036, *p* = 0.047 < 0.05, $\eta_{p}^{2}$ = 0.04. Girls in the senior class were more likely to trust teachers who provided material rewards than boys.

There was no age difference in the selective trust of boys towards teachers with material rewards, *F* (1, 109) = 1.515, *p* = 0.221, indicating that the level of trust in reward-giving teachers was the same for boys in the middle and senior classes. However, there was an age difference in the selective trust of girls towards teachers with material rewards, F (1, 109) = 6.503, *p* = 0.012 < 0.05, $\eta_{p}^{2}$ = 0.06. Girls in the senior class were more likely to trust teachers with material rewards than those in the middle class, with senior-class girls being more sensitive to material rewards.

The main effect of task type was not significant, *F* (1, 109) = 0.846, *p* = 0.36. The main effect of gender was not significant, *F* (1, 109) = 0.009, *p* = 0.924. The main effect of grade was not significant, *F* (1, 109) = 0.967, *p* = 0.328. This suggests that the development trend of selective trust towards teachers, accompanied by material rewards, is consistent among children in the middle and senior classes.

### 3.3. Discussion

Building on Experiment 1, Experiment 2 examined the impact of different types of teacher rewards on children’s selective trust, dividing teacher rewards into spiritual rewards and material rewards. Spiritual rewards refer to praise, while material rewards refer to physical gifts.

The study found that children in the junior class did not exhibit selective trust towards teachers who provided material rewards. Children begin to differentiate between praise and material rewards at the age of four, and children as young as three are more likely to trust teachers (Ma et al., 2017; Zhao, 2019). When faced with different types of rewards from teachers, all of which are positive and affirmative, there is little difference for children in the junior class, resulting in the same level of trust towards teachers with varying reward behaviors.

In the inquiry task, children in the senior class were more sensitive to teachers with material rewards than those in the middle class. In both tasks, senior-class girls were more likely to trust teachers with material rewards than middle-class girls, which is consistent with previous research. The form of rewards varies among different age groups, with children in the senior class being more enthusiastic about material rewards (Sun, 2008).

Additionally, senior-class girls were more likely to trust teachers who provided material rewards than senior-class boys. Our results are consistent with previous studies, indicating gender differences in children’s sensitivity to material rewards. Ding et al. (2017) found that under monetary reward conditions, boys had slower response times than girls, who showed higher sensitivity to monetary rewards. No gender differences were found in social feedback. This may be due to the gender differences in teacher praise observed in kindergartens, where girls receive praise more frequently than boys. Children perceive that, compared to boys, girls are more often praised by teachers, while boys receive less praise (Zhao, 2019). Therefore, for senior-class boys, the effects of praise and material rewards are similar, while girls may place more trust in the less frequently received material rewards.

Children from middle-class backgrounds were more likely to trust teachers with material rewards in the agreement task. This may be because the amount of information provided to children when making selective trust judgments is different in the two tasks. In the inquiry task, children only need to choose between material rewards and praise. In the agreement task, children not only need to consider the presence of material rewards but also the object’s name, which may increase the cognitive load on children, leading them to rely more on external material rewards to make judgments.

## 4. General Discussion

This study explored the impact of teacher rewards and their types on the selective trust of preschool children. The primary hypothesis was that preschool children would develop selective trust towards teachers who provide rewards, and that this trust would vary based on the type of reward (material vs. verbal) and the age and gender of the children. Specifically, it was hypothesized that material rewards would elicit stronger selective trust compared to verbal praise, and that younger children and girls would show higher levels of trust towards reward-providing teachers. The results of the study generally support the hypothesis that teacher rewards influence the selective trust of preschool children. Across different grades, children exhibited varying degrees of selective trust towards teachers who provided rewards. Children in the junior class showed the highest level of trust towards teachers who provided rewards, while children in the middle and senior classes also demonstrated trust, albeit to a lesser extent. In terms of reward types, children in the senior class, particularly girls, were more inclined to trust teachers who provided material rewards compared to those who offered verbal praise. These findings indicate that teacher rewards do have a significant impact on children’s selective trust, and that the type of reward and the demographic characteristics of the children play important roles in shaping this trust.

The results largely validate the initial hypothesis that teacher rewards enhance selective trust in preschool children. This study found that children across all grades preferred teachers who provided rewards over those who did not. This preference aligns with reinforcement theory, which suggests that rewards can reinforce appropriate behaviors in children, leading to positive emotional experiences and an improved teaching atmosphere (Hodges, 1972). The rewarding behavior of teachers, therefore, serves as a powerful tool for building trust. The findings also support the notion that younger children, specifically those in the junior class, are more sensitive to rewards. This heightened sensitivity may be attributed to their limited experience and familiarity with teachers, making them more susceptible to positive reinforcement (Weisz, 1977).

However, the results also revealed some inconsistencies with the initial assumptions. Not all preschool children exhibited selective trust towards teachers who provided material rewards. For instance, children in the junior class did not show a preference for teachers who provided material rewards over those who offered verbal praise. This suggests that younger children may not yet have fully developed the ability to differentiate between types of rewards and their implications for trustworthiness (Fantuzzo et al., 1991). The presence of rewards, regardless of their type, may be sufficient to evoke a sense of trust in some children, indicating that the trust observed may not be truly selective but rather a preference for reward-providing individuals (Alvarez & Booth, 2014; Csibra & Gergely, 2006; Stower et al., 2024). In the middle class, only boys exhibited selective trust towards teachers who provided material rewards. This gender difference may reflect varying sensitivity to rewards among boys and girls, influenced by their prior experiences and socialization processes (Zhao, 2019). Boys in the middle class are often more active and receive less praise and rewards from teachers, making them more sensitive to material rewards. In contrast, girls may have a more balanced perception of both material and verbal rewards.

In the senior class, children, especially girls, were more inclined to trust teachers who provided material rewards. This preference may be due to increased exposure to adults and societal influences as children grow older. The market economy, which often uses material interests to measure value, may shape children’s preferences, making them more inclined towards material rewards (Ding et al., 2017; Sun, 2008). This finding suggests that the type of reward and the child’s developmental stage play crucial roles in shaping selective trust.

While this study confirms that teacher rewards can influence children’s selective trust, it also highlights the complexity of this relationship. The findings suggest that rewards may enhance children’s trust in teachers, but they may not necessarily promote genuine selective trust based on informational reliability. Educators should be aware that the use of rewards may lead children to prefer reward-giving teachers without critically evaluating their reliability as informants. Future research should explore how teachers can balance the use of rewards with strategies that promote children’s critical evaluation skills, thereby fostering both trust and selective trust in educational contexts.

This study has several limitations. Firstly, it primarily explored the impact of teacher rewards on the selective trust of preschool children, focusing on rewards commonly used by teachers. From the children’s perspective, this was a passive acceptance process. Future research could consider rewards and praise that children prefer most to determine if they have a different impact on selective trust. Secondly, the study only examined the impact of positive factors such as rewards, specifically material rewards and praise. Future research could explore the impact of the frequency of teacher rewards, other types of rewards, or punishment on children’s selective trust to further enrich the understanding of how teacher behavior influences children’s trust. Additionally, the use of animated videos may have compromised ecological validity. Future research could enhance ecological validity by employing more natural methods, such as observing real classroom interactions or using live experimenter interactions, to capture the authenticity of teacher–child dynamics better and improve the generalizability of findings to real-world educational settings. Lastly, it remains unclear whether the neutral feedback used in this experiment might unconsciously influence children’s decisions, and whether children’s responses reflect preferences rather than a genuine evaluation of teacher credibility. Therefore, future designs should include a truly neutral teacher who provides no evaluative feedback to control for the reward effect, and incorporate interviews to explicitly assess children’s understanding of neutral feedback, thereby distinguishing epistemic trust from affective preference.

## 5. Conclusions

In summary, our study suggests that the rewarding behavior of teachers has a significant impact on the selective trust of preschool children, with those in the junior class exhibiting the highest sensitivity to teachers who provide rewards. Furthermore, different types of teacher rewards elicit varying levels of trust among children. While junior-class children exhibit equal trust towards teachers offering either material rewards or verbal praise, middle- and senior-class children, particularly senior-class girls, demonstrate selective trust towards teachers who provide material rewards, suggesting that tangible incentives may more influence older children. Educators should choose appropriate reward types and adjust strategies based on children’s developmental stages to foster both trust and critical thinking.

## Figures and Tables

**Table 1:** Participants’ basic information.

Kindergarten Grade	Gender (*n*)	Month of Age		Range	
		*M*	*SD*	*Min*	*Max*
Junior Class	Male (36)	45.65	3.26	40.10	51.67
	Female (27)	45.78	3.00	38.60	51.73
	All (63)	45.71	3.12	38.60	51.73
Middle Class	Male (25)	56.43	3.56	53.16	64.53
	Female (27)	55.78	2.35	53.40	62.10
	All (52)	56.09	2.98	53.17	64.53
Senior Class	Male (31)	70.97	2.98	66.46	76.67
	Female (23)	70.59	3.01	65.17	74.63
	All (54)	70.81	2.97	65.17	76.67

**Table 2:** Children’s inquiry degree and agreement degree towards reward-giving teachers.

Grade	Gender (*n*)	Inquiry Degree (*M* ± *SD*)	Agreement Degree (*M* ± *SD*)
Junior Class	Male (36)	0.24 ± 0.28	0.26 ± 0.31
	Female (27)	0.37 ± 0.19	0.35 ± 0.19
	All (63)	0.29 ± 0.25	0.30 ± 0.27
Middle Class	Male (25)	0.18 ± 0.28	0.25 ± 0.23
	Female (27)	0.22 ± 0.21	0.21 ± 0.23
	All (52)	0.20 ± 0.25	0.23 ± 0.23
Senior Class	Male (31)	0.21 ± 0.22	0.20 ± 0.25
	Female (23)	0.21 ± 0.28	0.18 ± 0.32
	All (54)	0.21 ± 0.25	0.19 ± 0.28

**Table 3:** Participants’ basic information.

Kindergarten Grade	Gender	Month of Age		Range	
		*M*	*SD*	*Min*	*Max*
Junior Class	Male (32)	44.07	4.01	37.03	50.50
	Female (31)	45.44	3.04	39.33	50.63
	All (63)	44.74	3.61	37.03	50.63
Middle Class	Male (26)	55.80	2.04	53.03	61.83
	Female (28)	55.99	3.24	52.77	63.30
	All (54)	55.90	2.71	52.77	63.30
Senior Class	Male (33)	70.58	2.77	66.20	75.73
	Female (26)	69.86	3.67	66.17	80.73
	All (59)	70.27	3.19	67.17	80.73

## Data Availability

The data supporting the findings of this study are available from the corresponding author on reasonable request.

## Appendix A. Preschool Teacher Praise and Reward Survey

1. Which class do you teach? (Single Choice)	1. 您所任教的班级是？（单选）
A. Nursery Class	A. 小班
B. Middle Class	B. 中班
C. Senior Class	C. 大班
2. Top four most frequently used phrases to praise children (Multiple Choice)	2. 最常使用的前四项表扬用语（多选）
A. You’re awesome!	A. 你真棒！
B. You’ve improved!	B. 你有进步！
C. You’re amazing!	C. 你真厉害！
D. You did great!	D. 你做得很好！
E. You’re smart!	E. 你真聪明！
F. Learn from him/her!	F. 大家要向他/她学习！
G. You’re our role model!	G. 你是大家的小榜样！
H. You’re sensible!	H. 你真懂事！
I. You’re obedient!	I. 你真听话！
J. You’re outstanding!	J. 你真优秀！
3. Top four most frequently used rewards for children (Multiple Choice)	3. 最常使用的前四项奖励物品（多选）
A. Little Red Flower	A. 小红花
B. Small Stickers	B. 小贴纸
C. Stickers	C. 贴纸
D. Star Stickers	D. 小星星贴纸
E. Certificate	E. 奖状
F. Small Toys	F. 小玩具
G. Candy	G. 糖果
H. Colored Clay	H. 彩泥
I. Crayons	I. 蜡笔
J. Handmade Gifts	J. 手工小礼物
4. Top four phrases praising children’s effort (Multiple Choice)	4. 表扬幼儿努力程度的前四项用语（多选）
A. You’re diligent!	A. 你真勤奋！
B. You’re resourceful!	B. 你点子真多！
C. You’re thoughtful!	C. 你考虑得真周到！
D. You’re attentive!	D. 你真专心！
E. You’re hardworking!	E. 你特别用功！
F. You’re observant!	F. 你观察真仔细！
G. You’re curious!	G. 你很好奇！
H. You love learning!	H. 你爱学习！
I. You’re persistent!	I. 你坚持到底！
J. You’re studious!	J. 你好学！
5. Top four phrases praising children’s ability (Multiple Choice)	5. 表扬幼儿能力的前四项用语（多选）
A. You’re capable!	A. 你真能干！
B. You’re impressive!	B. 你真让人惊喜！
C. You’re awesome!	C. 你真了不起！
D. You’re smart!	D. 你真聪明！
E. You’re a good kid!	E. 你是个好孩子！
F. You’re sensible!	F. 你真懂事！
G. You’re adorable!	G. 你真可爱！
H. You’re obedient!	H. 你真听话！
I. You’re excellent!	I. 你太出色了！
J. You’re clever!	J. 你真机灵！
6. Top four activities that give children a sense of achievement (Multiple Choice)	6. 最能给予幼儿成就感的前四项活动（多选）
A. Handicrafts	A. 手工制作
B. Building Blocks	B. 积木搭建
C. Drawing	C. 绘画
D. Puzzles	D. 拼图游戏
E. Singing	E. 唱歌
F. Role Play	F. 角色扮演
G. Storytelling	G. 讲故事
H. Dancing	H. 跳舞
I. Climbing Slides	I. 滑滑梯
J. Soccer	J. 踢足球

## Appendix B. Survey Results on Preschool Teachers’ Praise and Reward Methods

**Category**	**Item**	**Percentage (%)**	**Percentage**
Verbal Praises	“You’re awesome!”	73.2	30
	“You’ve improved!”	63.4	26
	“You’re amazing!”	51.2	21
	“You performed well!”	46.3	21
Reward Items	Red flowers	82.9	34
	Stickers	63.4	26
	Stars	51.2	21
	Certificates	46.3	19
Achievement Activities	Craft-making	68.3	28
	Block-building	61.0	25
	Drawing	58.5	24
	Puzzle-solving	51.2	21
